# When leadership turns covert: conceptualizing and developing a scale for Leader’s Manipulative Workplace Behavior

**DOI:** 10.3389/fpsyg.2026.1803662

**Published:** 2026-07-08

**Authors:** Dongying Luo, Zheyuan Wang

**Affiliations:** 1 School of International Business, Zhejiang Yuexiu University, Shaoxing, Zhejiang, China; 2 School of Labor and Human Resources, Renmin University of China, Beijing, China

**Keywords:** destructive leadership, gaslighting, Leader’s Manipulative Workplace Behavior, psychological manipulation, scale development

## Abstract

This research conceptualizes and operationalizes Leader’s Manipulative Workplace Behavior (LMWB)—defined as a leader’s conscious attempt to alter a subordinate’s perception of reality and self-concept to achieve mental control and fulfill selfish goals. A scale measuring the LMWB was developed and validated through multiple empirical samples. In Study 1, items were developed, and their content validity was assessed through two samples of subject matter experts. In Study 2, following item analysis and an initial exploratory factor analysis (EFA) on Sample 2a (*N* = 249), a 10-item scale was developed. Subsequently, Sample 2b (*N* = 695) was used in the second EFA to verify the validity of the retained items. In Study 3, Sample 3a (*N* = 200) was used for confirmatory factor analysis (CFA) and reliability testing, and Sample 3b (*N* = 256) was used to assess convergent validity, discriminant validity, nomological network, predictive validity, and incremental validity. The results confirmed the scale’s reliability and validity and demonstrated that LMWB uniquely predicts employee outcomes beyond the effects of abusive supervision and exploitative leadership. This study provides the first measurement tool for this covert form of psychological manipulation and discusses its significant theoretical and practical implications.

## 1 Introduction

In contemporary workplaces, a pervasive yet under-studied form of psychological distress is emerging, stemming not from overt hostility but from subtle leader manipulation. A 2023 survey by Zhaopin, a leading Chinese recruitment platform, paints a startling picture: 84.9% of respondents reported experiencing workplace injustice, and 50.8% specifically identified themselves as victims of leader manipulation. These manipulative behaviors, characterized by gaslighting and implicit coercion, create a climate of fear and self-doubt in employees, leading to emotional distress and diminished productivity. This phenomenon aligns with broader concerns about interpersonal mistreatment at work (Bowling and Beehr, 2006; Leake et al., 2025) and the significant costs of toxic leadership to organizational health (Tepper, 2007; Bhattacharjee and Sarkar, 2024). Despite its perceived prevalence, academic research on this phenomenon remains fragmented and inadequately captured by existing destructive leadership constructs. This gap is particularly evident when considering the literature on psychological manipulation and gaslighting, which has documented similar interpersonal dynamics but is yet to be systematically applied to the leader-subordinate context.

Over the past two decades, the field of organizational behavior has explored the “dark side” of leadership, predominantly through constructs such as abusive supervision—defined as subordinates’ perceptions of the extent to which supervisors engage in the sustained display of hostile verbal and non-verbal behaviors, excluding physical contact (Tepper, 2000)—and, more recently, exploitative leadership, which captures leaders’ self-serving practices that leverage oppression for personal gain (Schmid et al., 2019). These foundational constructs, along with broader frameworks of destructive leadership (Krasikova et al., 2013; Korkmazyurek and Ocak, 2024), have been invaluable in highlighting the detrimental effects of overtly hostile (Chi and Liang, 2013; Gu et al., 2016; Mackey et al., 2017; Lee et al., 2020) or manifestly self-interested leadership actions (Abdulmuhsin et al., 2021). However, existing research mainly focuses on explicit and easily perceived behaviors such as belittlement, ridicule, public aggression, and stealing credit from subordinates (Keashly et al., 1994; Tepper, 2000; Schyns and Schilling, 2013). This focus has resulted in critical oversight in understanding subtle and psychologically manipulative behaviors that are equally pervasive in the workplace. The significant harm wrought by such covert and indirect aggression is well-established in social psychology, recognized as a distinct and insidious form of interpersonal mistreatment (Björkqvist, 1994; Williams, 2007). This further highlights the necessity of conducting in-depth research on subtle yet harmful workplace dynamics. This oversight has been further underscored by research on covert aggression and psychological manipulations. Unlike overt hostility, covert manipulation operates through deceptive and indirect tactics designed to control another person’s beliefs, emotions, and behaviors without conscious awareness (Susser et al., 2019). In organizational settings, subtle forms of interpersonal mistreatment—such as undermining, social exclusion, and gaslighting—have been shown to cause significant psychological harm that often exceeds that of overt aggression (Kukreja and Pandey, 2023; Popat and Pandey, 2026). However, these insights from the literature on manipulation have rarely been integrated into the study of leadership behaviors, leaving a conceptual void in understanding how leaders employ subtle psychological tactics to achieve mental control over their subordinates.

To address this gap, we conceptualize “Leader’s Manipulative Workplace Behavior” (LMWB) as a pattern of covert, indirect, and psychologically controlling behaviors employed by leaders to influence employees’ thoughts, affects, and behaviors for their own purposes. Specifically, LMWB is rooted in the literature on psychological manipulation and gaslighting (Roberts and Andrews, 2013; Davis and Ernst, 2019; Suskind, 2023; Johnson et al., 2021), which identifies patterns of covert interpersonal control involving deception, exploitation of vulnerabilities, and influence tactics that bypass rational decision-making and describes a manipulative leadership style characterized by strategic deceit and self-serving influence. Recent empirical work by Kukreja and Pandey (2023) operationalized gaslighting in the workplace. They developed a 12-item Gaslighting at Work Questionnaire (GWQ) and identified gaslighting as a second-order construct comprising two dimensions: trivialization (undermining subordinates’ perspectives, fears, and realities) and affliction (directing emotional pain toward subordinates). However, LMWB is distinct from workplace gaslighting in two important respects. First, while gaslighting focuses primarily on making targets doubt their own sanity through trivialization and affliction, the LMWB encompasses a broader repertoire of tactics. In addition to reality distortion, it employs illusory promise-making and false flattery—tactics that do not necessarily inflict overt pain but are designed to secure compliance through a manipulated self-concept. Second, the defining goal of LMWB is not merely psychological destabilization but the establishment of mental control and long-term obedience. Translating this dynamic into the leader-subordinate hierarchy, we finally define LMWB as a leader’s conscious attempt to alter a subordinate’s perception of reality and self-concept to achieve mental control and fulfill selfish goals. In addition, it is critical to distinguish LMWB from related constructs to establish its conceptual uniqueness. Unlike the hostile negativity of abusive supervision (Tepper, 2000), the LMWB is characterized by its insidious and calculated nature. Furthermore, while exploitative leadership (Schmid et al., 2019) shares an instrumental and self-serving core with LMWB, it often manifests as overtly oppressive and deceptive acts, such as making unreasonable demands or taking credit for subordinates’ work. In contrast, LMWB operates through a more subtle and psychological process, which means that the purpose of LMWB is not necessarily to squeeze employees but to make employees obey. Based on the above analysis, the detrimental impact of LMWB is hypothesized to be profound and distinct from that of overt abuse. Eroding employees’ sense of self-efficacy and consuming cognitive resources through constant coping and sense-making can lead to psychological exhaustion. For organizations, this behavior likely fosters a culture of silent compliance, superficial conformity, and suppressed psychological safety, thereby inhibiting innovation. However, the lack of a valid and reliable measurement tool severely hinders the systematic empirical investigation of these mechanisms and consequences.

**Figure 1:**
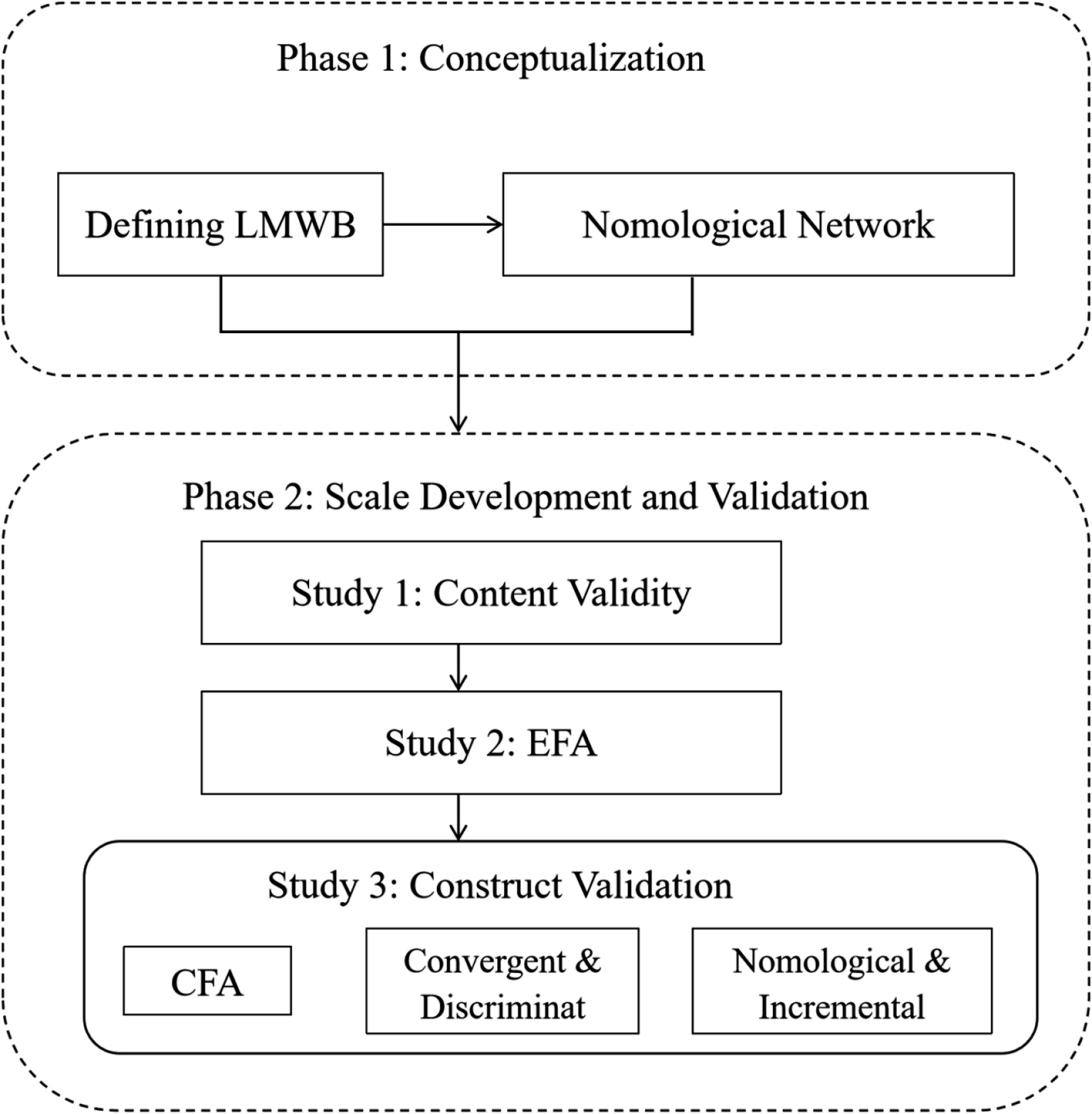
Conceptual framework figure.

Therefore, we develop and validate a scale for LMWB, provide evidence of our scale’s content and discriminant validity, and demonstrate its incremental predictive validity of employee attitudes and behaviors above and beyond other leadership constructs (such as abusive supervision and exploitative leadership). Figure 1 presents the overarching conceptual framework that guides our research and illustrates the logical connections among the sub-studies. In doing so, we make several contributions to the literature. First, current research on improper leadership behavior mainly focuses on overt hostility or authoritative suppression (Lee et al., 2020), while lacking systematic identification and theoretical refinement of more covert and psychologically manipulative behaviors. By introducing the concept of LMWB, we extend this field to deepen from overt behaviors to hidden psychological mechanisms, responding to the academic call for a more detailed distinction between different types of negative leadership behaviors (Mitchell et al., 2023). LMWB captures a distinct and insidious form of manipulation that explains additional variance in a range of employee outcomes—including reduced trust and well-being, heightened emotional exhaustion, diminished performance, and increased turnover intention—above and beyond the effects of abusive supervision and exploitative leadership. Second, we develop and validate a scale to measure LMWB. The scale developed in this study makes it possible to conduct a quantitative analysis of large-scale and diverse samples in future research, thereby enhancing the generalizability and practical applicability of the research. Third, by grounding LMWB in the psychological manipulation and workplace gaslighting literature, we bridge the previously disconnected streams of research. This scale fills this methodological gap, provides a reliable tool for the systematic testing of LMWB in research on organizational and leadership behavior, effectively helps us grasp the structure, antecedents, and consequences of this behavior, and lays the foundation for subsequent empirical connections with other variables.

## 2 Conceptualization of Leader’s Manipulative Workplace Behavior

### 2.1 Defining “Leader’s Manipulative Workplace Behavior”

The theoretical foundation of Leader’s Manipulative Workplace Behavior (LMWB) rests primarily on the literature of gaslighting and psychological manipulation in interpersonal and organizational contexts. Gaslighting originated as a clinical concept describing a pattern of psychological abuse in which the perpetrator systematically distorts the target’s reality to induce self-doubt, confusion, and dependency (Roberts and Andrews, 2013; Davis and Ernst, 2019, Suskind, 2023; Johnson et al., 2021). Over time, the concept has been extended beyond intimate relationships to capture manipulative dynamics in various hierarchical settings. The inherent power imbalance in organizations makes this environment particularly susceptible to such tactics, which evolve into a unique and pernicious form of psychological manipulation. In order to more accurately describe the nature and connotation of workplace PUA behavior, our study conceptualizes it as “Leader’s Manipulative Workplace Behavior” (LMWB) and defines it as a leader’s conscious attempt to alter a subordinate’s perception of reality and self-concept to achieve mental control and fulfill selfish goals. This definition underscores that LMWB is not merely about overt confidence attacks but encompasses insidious behaviors designed to distort an employee’s cognitive and emotional landscape. In practice, these behaviors include instilling alternative narratives to reshape subordinates’ beliefs, employing calculated praise or isolation to foster dependency, using implicit threats to induce compliance, and imposing specific labels to pressure subordinates into conforming roles. While behaviorally diverse, these tactics are theorized as unified indicators of a single underlying latent trait: the leader’s intention is to achieve control by systematically distorting the perceptions of subordinates. This supports our conceptualization of LMWB as a unidimensional construct focused on psychological control.

### 2.2 Nomological network

To fully establish the theoretical distinctiveness of Leader’s Manipulative Workplace Behavior (LMWB), this section delineates its nomological network by clarifying its conceptual boundaries, identifying key antecedents, and specifying its likely outcomes. First, we differentiate LMWB from two major established constructs of destructive leadership—abusive supervision and exploitative leadership—to highlight its unique theoretical space. Next, we outline the antecedents that may predispose leaders to LMWB. Finally, based on emerging qualitative and theoretical studies, we summarize the detrimental effects of LMWB on employee well-being and performance. Together, this network positions LMWB as a distinct form of covert psychological manipulation with its own theoretical and practical significance.

#### 2.2.1 Abusive supervision

Research on abusive supervision reveals its complex, multilevel nature (Schyns and Schilling, 2013; Mullen et al., 2018; Priesemuth and Schminke, 2019). Scholars have defined it as “subordinates’ perceptions of the extent to which supervisors engage in the sustained display of hostile verbal and non-verbal behaviors, excluding physical contact” (Tepper, 2000, p. 178), encompassing behaviors like verbal attacks, intimidation, and unfair treatment (Hu and Liu, 2017). This phenomenon can produce a “double-edged sword” effect depending on subordinate attributions (Liao et al., 2021) and can cascade through organizations, as middle managers may imitate the abusive behaviors of senior leaders based on their interpretation of top management’s intentions (Liu et al., 2012). Notably, such behavior may even offer short-term psychological recovery for the perpetrators themselves, although this effect is transient and conditional (Qin et al., 2018). Within this established context, we introduce LMWB as a distinct yet related construct. LMWB and abusive supervision intersect in certain respects but are also fundamentally different. In terms of similarities, first, the two leaders’ behaviors were implemented to achieve their goals. Second, LMWB shares similarities with the manifestations of abusive supervision; they can both show hostility toward subordinates and use direct negative means to influence them. However, critical differences define their unique theoretical spaces. First, in terms of purpose, while abusive supervision may sometimes stem from a leader’s frustration or a misguided belief in “tough love” to motivate employees and push them to improve, LMWB is fundamentally and instrumentally driven by the leader’s self-interest. Second, compared with abusive supervision, the manifestations of LMWB are more diversified. In addition to the means of expressing hostility, LMWB may take the form of illusory promise-making and more positive verbal means, such as flattering employees to create a wrong perception of themselves. Third, the core of LMWB lies in mental control; that is, they need to change employees’ original cognition and make them obey it, while abusive supervision does not have this feature. Thus, while they intersect, LMWB represents a more insidious and psychologically complex form of interpersonal harm in the workplace.

#### 2.2.2 Exploitative leadership

Exploitative leadership is a new destructive leadership structure proposed in recent years that refers to the process by which leaders achieve their personal goals by means of pressure, manipulation, and hindering the development of subordinates. It is essentially egoism and uses subordinates to achieve personal goals (Schmid et al., 2019). Studies have found that exploitative leadership will increase burnout, workplace bias, alienation, and Counterproductive Work Behavior (CWB) of subordinates (Schmid et al., 2019; Lyu et al., 2023), and it will also hinder employees’ knowledge-sharing behavior (Abdulmuhsin et al., 2021).

Schmid et al. (2019) divided it into five dimensions: (i) genuine egoistic behaviors, which means that leaders do not regard employees as individuals but as tools to ensure the completion of their own goals; (ii) exerting pressure on employees, which means that the leader in the organization completely disregards the employees’ work pressure when pursuing personal goals, such as “Increases my workload without considering my needs in order to reach his or her goals”; (iii) undermining development, which means that the leader deliberately hinders the employees’ growth for their personal gain, such as “Does not give me opportunities to further develop myself professionally because his or her own goals have priority”; (iv) taking credit for others’ efforts, which means that the leader takes the fruits of the employees for himself/herself, such as “Uses my work to get himself or herself noticed”; and (v) manipulative followers, which means that the leader achieves personal goals through tactics such as spreading rumors and deceiving, such as “Plays my colleagues and me off against each other to reach his or her goals.”

Our study points out that although exploitative leadership and LMWB both have self-serving purposes, exploitative leaders mainly regard employees as a means to achieve their goals, while LMWB focuses on spiritual manipulation of employees; that is, the purpose of LMWB is not necessarily to exploit employees for maximum output but to make employees obey. For example, leaders may falsely flatter employees so that they have an incorrect understanding of themselves and must follow or obey the instructions of leaders after a failure or error.

#### 2.2.3 Antecedents

The propensity of leaders to engage in LMWB is not casually distributed; it is systematically influenced by dispositional traits and psychological states. First, individuals with higher empathy and agreeableness (Chapman et al., 2007) are quicker to empathize with others at work, more helpful, and tolerant, and therefore less likely to implement LMWB on people around them. Conversely, individuals with high Machiavellianism—particularly on dimensions reflecting a desire for control and a propensity for unethical manipulation—are more likely to view LMWB as a viable tactic for achieving self-serving goals (Sarwar et al., 2023). Finally, the psychological state of power perception acts as a critical catalyst. Power perception refers to individuals’ psychological perception of privileges (Yam et al., 2017). When individuals perceive themselves as powerful, they tend to take their power for granted and feel entitled to prioritize their own interests. Consequently, they actively pursue outcomes that serve their interests. In turn, this leads to exploitation and non-altruistic behaviors such as LMWB.

#### 2.2.4 Employee outcomes

Current research does not carry out a quantitative analysis of LMWB; only a few Chinese scholars have theoretically expounded on the factors influencing leadership workplace manipulation behaviors in Chinese journals. For example, Huang et al. (2023) pointed out that workplace PUA in the medical community will impact the physical and mental health of medical workers (such as anxiety, depression, post-traumatic stress disorder, and chronic diseases), personal interests, and career prospects (such as increased turnover intention caused by unfair treatment). In addition, as healthcare workers are affected by LMWB, there will be further impacts on patients (such as medication errors caused by the mental distraction of healthcare workers after bullying). Similarly, some healthcare workers subjected to LMWB ultimately leave their jobs, undermining overall team stability. The frequent recruitment of replacements and associated training further compromises the team’s overall work efficiency. In the context of LMWB, individuals’ self-confidence, self-esteem, and sense of self-worth are destroyed. Trapped in this situation, individuals may be in a state of self-debasement, depression, or even suicide.

## 3 Development and validation of the LMWB scale

Next, we describe our process, following Cortina et al. (2020), Hinkin (1995), and recent scale development studies (e.g., Jean et al., 2024). The specific steps are as follows: First, we constructed a clear description that defines the nature of LMWB and its differentiation from other concepts. Second, we generated items following an inductive approach, as no prior literature has systematically analyzed this construct. Finally, we assessed the initial items for content validity, reliability, discriminant and convergent validity, nomological network validity, and predictive and incremental validity and conducted exploratory and confirmatory factor analyses.

### 3.1 Study 1: content validation method and results

#### 3.1.1 Item generation

Since there is no systematic research on LMWB, our study followed the inductive method described by Hinkin (1995) to generate constructs. Specifically, our study initially conducted in-depth interviews with 12 employees from diverse enterprises in the healthcare, information technology, and education sectors. Participants held positions ranging from entry-level to middle management, with organizational tenure ranging from 1 to 12 years. Each semi-structured interview lasted for approximately 30–45 min. To supplement interviews, we also analyzed “Workplace Pickup Artist (PUA)” discussions on Zhihu (556 replies) until reaching thematic saturation. It should be noted that “Workplace PUA” was used as a culturally familiar prompt during interviews and as a search term for online data collection, whereas LMWB served as the formal conceptual reference for coding. Through interviews and Zhihu Content Analysis, behavioral statements were extracted and consolidated through open coding and axial categorization, with redundant statements merged through research team discussion. This process generated 19 items designed to capture leaders’ psychologically manipulative behaviors. Two authors independently verified that each of the 19 items fully covered the content of the construct (see Appendix A). The final items are presented in Appendix B.

The LMWB items were originally developed in Chinese. One author translated the items into English, and an independent bilingual scholar back-translated them into Chinese. The two authors compared the back-translated version with the original version to ensure conceptual equivalence.

#### 3.1.2 Content validation

First, 14 experts familiar with workplace misconduct were invited to make conceptual judgments on LMWB. The 19 items were scored on a scale of 5 levels ranging from very irrelevant to very relevant based on their correlation with the corresponding concept. At the same time, experts were required to supplement the related behaviors of LMWB to further ensure that the collected items reached theoretical saturation. The results showed that the 19 items collected through interviews and responses to Zhihu met the requirements of theoretical saturation. Then, the content validation was conducted using an item classification task (Brady et al., 2017). In this study, 6 professors were invited to categorize 19 items in the LMWB and two similar concepts (abusive supervision and exploitative leadership). After categorization, the degree of association was scored from 1 (poor fit with the definition) to 5 (strong fit with the definition). To avoid bias, our study only provides explanations for these concepts. Referring to the research results of Brady et al. (2017), when the items were classified more frequently under the concept of LMWB than under other concepts and the item-level content validity index (I-CVI) was higher than 0.78, it was considered that the item had passed. The formula for calculating the content validity index is the number of experts who chose options 4 and 5 for each item divided by the total number of experts. Our results showed that all 19 items had strong content validity, with an average scale-level content validity index (S-CVI) of 0.94. Although item-level misclassification frequencies were not systematically recorded in this categorization task, the high S-CVI indicates that experts consistently rated the 19 items as highly relevant to LMWB. Therefore, we retained 19 items.

### 3.2 Study 2: EFA method and results

#### 3.2.1 Participants

Our study used the Credamo platform to recruit participants. After collecting 260 questionnaires, we eliminated samples that had too short or too long response times and manually reviewed each questionnaire. Eventually, we accepted 249 questionnaires (Sample 2a in Table 1), and the respondents would receive a reward of 5. The participants were 74 males (29.7%) and 175 females (70.3%). Regarding age, 79 people were in the 20–30 age group (accounting for 31.7%), 163 people were in the 31–40 age group (65.5%), 3 people were in the 41–50 age group (1.2%), 2 people were in the 51–60 age group (0.8%), and 2 were over 60 years old (0.8%). Regarding education, 12 people had an education level below a bachelor’s degree (4.8%), 140 people had a bachelor’s degree (56.2%), 92 people had a master’s degree (37.0%), and 5 people had a doctoral degree (2.0%). The participants had an average of 6.46 years of work experience.

**Table 1:** Overview of study phases and samples.

Study description	Samples used
Study 1: content validation: Item generation and content validation	1a,1b,1c
Study 2: EFA method	2a, 2b
Study 3: construct validation method and results	3a,3b

Sample description	*N*
Sample 1a: interviewers	12
66.67% female, 83.33% master’s degree, 16.67% bachelor’s degree	
Sample 1b: experts	14
50% female, 28.57% professors, 42.86% assistant professors, 28.57% associate professors, 100% doctoral degree	
Sample 1c: professors	6
16.67% female; 53 average age	
Sample 2a EFA1	249
70.3% female; age: 31.7% in 20–30, 65.5% in 31–40, 1.2% in 41–50, 0.8% in 51–60, 0.8% over 60; 2.0% doctoral degree, 37.0% master’s degree, 56.2% bachelor’s degree, and 4.8% below; 6.46 average working years	
Sample 2b EFA2	695
41.3% female; age: 44.2% in 20–30, 37.4% in 31–40, 13.4% in 41–50, 4.9% in 51–60, 0.1% over 60; 10.1% doctoral degree, 48.8% master’s degree, 28.9% bachelor’s degree, and 12.2% below; working years:15.3% less than 1, 36.1% in 1–3, 17.2% in 4–6, 9.8% in 7–10, and 21.6% more than 10	
Sample 3a CFA	200
55.3% female; age: 30.5% in 20–30, 36% in 31–40, 26% in 41–50, and 7.5% in 51–60; 10% doctoral degree, 29% master’s degree, 56% bachelor’s degree, and 5% below; working year: 6% less than 1, 48.5% in 1–3, 16.5% in 4–6, 14% in 7–10, and 15% more than 10	
Sample 3b	256
30.5% female; age: 16.4% in 20–30, 38.7% in 31–40, 29.3% in 41–50, 9.8% in 51–60; and 12.5% doctoral degree, 47.7% master’s degree, 26.2% bachelor’s degree, and 13.7% below; working year: 12.9% less than 1, 29.3% in 1–3, 28.9% in 4–6, 9.8% in 7–10, and 19.1% more than 10	

#### 3.2.2 Measures

The participants rated the extent to which they agreed with each of the 19 LMWB items on a 7-point Likert scale with anchors of 1 (strongly disagree) to 7 (strongly agree).

#### 3.2.3 Results

##### 3.2.3.1 Item analysis results

In order to preliminarily test the discrimination of each item in the scale, we first used the Statistical Package for the Social Sciences (SPSS, Version 26.0; IBM Corp., NY, USA) to conduct item analysis on the LMWB scale. Following the recommendations of Wu (2009), all 19 items met both criteria, demonstrating strong discrimination. In addition, we conducted a reliability analysis. The internal consistency coefficient of the LMWB scale was 0.97, and deleting any item did not increase the reliability of the scale.

##### 3.2.3.2 Exploratory factor analysis

To confirm whether the questionnaire is suitable for factor analysis, we first conducted the Kaiser–Meyer–Olkin (KMO) test and Bartlett’s test of sphericity. Through the analysis, the KMO value of LMWB was 0.969, Bartlett’s test value was 4591.049, *p* < 0.001, and the commonality of each item was greater than 0.3. Thus, the results indicated that the data were suitable for conducting exploratory factor analysis. We used a factor analysis function with a principal component analysis and orthogonal rotation. Based on the item deletion standard of previous research (e.g., Hu and Gan, 2008), we found that nine items did not meet the standards, exhibiting issues such as cross-loadings and low factor loadings. Through rigorous analysis, we decided to delete these nine items. Considering that there were deleted items, our study reanalyzed the items for discrimination. The steps followed the same process as in the first item analysis. The results showed that 10 items had significant discrimination. At the same time, the KMO value after deletion was 0.939, Bartlett’s test value was 1953.716, and *p* < 0.001, which are suitable for factor analysis. Then, an exploratory factor analysis was conducted again on these 10 items. The results showed that there was only 1 factor with an eigenvalue greater than 1, and the cumulative variance contribution rate was 64.92%. Previous studies have shown that extracting factors that explain more than 60% of the total variance meets the standard. Thus, we retained 10 items for additional analyses.

##### 3.2.3.3 Second exploratory factor analysis

To further ensure the accuracy of item elimination, we conducted a second round of exploratory factor analysis of the revised LMWB scale using Sample 2b. After removing invalid responses from the 700 employees initially recruited across regions such as Beijing, Shanghai, Shandong, and Jiangsu, 695 valid questionnaires were retained for subsequent analysis. Before the formal analysis, a descriptive statistical analysis was conducted on Sample 2b. Among the 695 valid data, 408 were male (accounting for 58.7%), and 287 were female (accounting for 41.3%). By age, 307 were between 20 and 30 years old (accounting for 44.2%), 260 were between 31 and 40 years old (37.4%), 93 were between 41 and 50 years old (13.4%), 34 were between 51 and 60 years old (4.9%), and 1 was over 60 years old (0.1%). Regarding education, 85 had a bachelor’s degree (12.2%), 201 had a bachelor’s degree (28.9%), 339 had a master’s degree (48.8%), and 70 had a doctoral degree (10.1%). For working years, 106 had less than 1 year (15.3%), 251 had 1–3 years (36.1%), 120 had 4–6 years (17.2%), 68 had 7–10 years (9.8%), and 150 had more than 10 years (21.6%). The factor analysis results showed that the KMO value was 0.950, Bartlett’s spherical test value was 11864.313, and *p* < 0.001. Each item’s commonality was greater than 0.3, which met the standards for factor analysis. Following the steps of the first exploratory factor analysis, principal component analysis and orthogonal rotation were used to extract factors with eigenvalues greater than 1. The final results are shown in Table 2. The revised 10-item LMWB scale had a single-factor structure, with a cumulative variance explanation rate of 83.34%. After revision and editing, cross-loading of the scale improved significantly, indicating that deletion during the revision process was necessary. Therefore, we retained 10 items for additional analyses.

**Table 2:** Results of exploratory factor analysis for the final 10 Items.

Item	Factor
Deliberately emphasizing his/her importance to you to make you follow.	0.84
Deliberately instilling his/her thoughts into you to unconsciously change your cognition.	0.81
Intentionally seeking out flaws in your thinking to make you follow him/her.	0.91
Making empty promises in your work to make you follow his/her orders.	0.88
Deliberately sabotaging your relationships with others to isolate you and make you ultimately dependent on him/her.	0.95
Deliberately showing hypocritical concerns to make you feel grateful and willing to obey him/her.	0.95
Deliberately emphasizing his/her power and status to make you obey him/her.	0.95
Threatening and intimidating you to make you have no choice but to obey.	0.84
Intentionally setting up a persona or label for you to make you act in accordance with it.	0.94
Deliberately emphasizing his/her importance to you to make you follow.	0.94

LMWB, Leader’s Workplace Manipulation Behavior.

### 3.3 Study 3: construct validation method and results

#### 3.3.1 Study 3a: confirmatory factor analysis

Next, we conducted a confirmatory factor analysis on LMWB to ensure the stability of structure development. A total of 200 valid questionnaires were collected, and we used Mplus 7.0 to analyze the LMWB scale.

##### 3.3.1.1 Participants

Among the 200 participants, 90 are male (45%), and 110 are female (55.3%). Regarding age, 61 are in the 20–30 age group (accounting for 30.5%), 72 are in the 31–40 age group (36%), 52 are in the 41–50 age group (26%), and 15 are in the 51–60 age group (7.5%). Regarding education, 10 are below a bachelor’s degree (5%), 112 have a bachelor’s degree (56%), 58 have a master’s degree (29%), and 20 have a doctoral degree (10%). For working years, 12 are less than 1 year (6%), 97 are in the 1–3 year range (48.5%), 33 are in the 4–6 year range (16.5%), 28 are in the 7–10 year range (14%), and 30 are more than 10 years (15%).

##### 3.3.1.2 Results

According to the standard in the existing literature (e.g., Wen et al., 2004), when *χ*^2^/df < 3, Root Mean Square Error of Approximation (RMSEA) < 0.08, Comparative Fit Index (CFI) > 0.90, and Tucker–Lewis Index (TLI) > 0.90, the model fits well. Our analysis showed that all indicators met the standards (results are shown in Table 3), indicating that the single-factor model had a good model fit.

**Table 3:** Confirmatory factor analysis of LMWB.

Model	*χ* ^2^	df	△*χ*^2^	*χ*^2^/df	RMSEA	CFI	TLI
A one-factor model	4.383	2		2.192	0.077	0.997	0.991

##### 3.3.1.3 Reliability test

Our study also utilized 200 questionnaires from Sample 3a to conduct an internal consistency assessment of LMWB. The results indicated that the internal consistency of LMWB was good, with a Cronbach’s *α* value of 0.937, which met the standard of *α* > 0.7.

#### 3.3.2 Study 3b: convergent and discriminant validity

We examined the correlations between LMWB, abusive supervision, and exploitative leadership. We used Sample 3b to conduct convergent and discriminant validity tests on the LMWB scale. Using correlation coefficients and average variance extracted (AVE) analysis, we examined whether LMWB was related to but distinct from these two constructs.

##### 3.3.2.1 Participants

We distributed questionnaires to full-time employees in Beijing. The participants who completed the first survey were invited to complete the second part 2 weeks later. A total of 270 responses were received (96.4% response rate). After eliminating invalid questionnaires, 256 valid questionnaires were obtained. Specifically, 178 were male (69.5%) and 78 were female (30.5%). Regarding age, 42 were between 20 and 30 years old (accounting for 16.4%), 99 were between 31 and 40 years old (38.7%), 75 were between 41 and 50 years old (29.3%), 25 were between 51 and 60 years old (9.8%), and 15 were over 60 years old (5.9%). Regarding education, 35 had a bachelor’s degree (13.7%), 67 had a bachelor’s degree (26.2%), 122 had a master’s degree (47.7%), and 32 had a doctoral degree (12.5%). For working years, 33 had less than 1 year (12.9%), 75 had 1–3 years (29.3%), 74 had 4–6 years (28.9%), 25 had 7–10 years (9.8%), and 49 had more than 10 years (19.1%).

##### 3.3.2.2 Measures

Except for the LMWB scale, which was independently developed in this research, all other variables were measured using mature scales, and these two variables were used with a 5-point Likert scale with anchors of 1 (strongly disagree) to 5 (strongly agree).

###### 3.3.2.2.1 Abusive supervision (T1)

Abusive supervision used the 15-item measurement items developed by Tepper (2000); an example item is “My leader is rude and disrespectful to me.” The Cronbach’s *α* coefficient was 0.99.

###### 3.3.2.2.2 Exploitative leadership (T1)

Exploitative leadership used the 15-item measurement items developed by Schmid et al. (2019); an example item is “My leader regards employees as tools to achieve personal goals.” Cronbach’s α was 0.99.

###### 3.3.2.2.3 Additional variables (T2)

We collected additional variables from this sample, which are used in the next section. These variables are discussed later in this article.

##### 3.3.2.3 Results

The results in Table 4 show that LMWB had different correlations with other types of leadership behaviors. LMWB was significantly correlated with abusive leadership (*r* = 0.551, *p* < 0.01) and exploitative leadership (*r* = 0.576, *p* < 0.01), indicating a moderate correlation.

**Table 4:** Correlation analysis of LMWB with other similar variables.

Variable	M	SD	1	2	3
1. LMWB	4.132	1.666	1		
2. Abusive supervision	2.928	1.224	0.551^**^	1	
3. Exploitative leadership	2.931	1.232	0.576^**^	0.937^**^	1

*N* = 256, ^**^p < 0.01, *p < 0.05, M = Mean, SD = Standard Deviation.

Based on the above analysis, we analyzed the discriminant validity and convergent validity of LMWB. Average variance extracted (AVE) is one of the indicators for measuring convergent validity. This reflects the explanatory power of the variance in the measured variable items. The higher the AVE, the higher the reliability and convergent validity of the construct to which it belongs. Generally speaking, AVE > 0.5 is considered to be in line with the standard. Composite Reliability (CR) reflects whether the measured variable items can consistently explain the variable. If CR > 0.7, it indicates good construct reliability. AVE and CR values of LMWB are shown in Table 5. The CR of LMWB is 0.9814, and the AVE is 0.8408. Both meet the standard, indicating good convergent validity of LMWB. Scholars have pointed out that when a variable’s square root is greater than the correlation coefficient between that variable and other variables, the discriminant validity of the construct is acceptable (Fornell and Larcker, 1981). Therefore, the square root result of the AVE of LMWB was compared with the correlation coefficients of other variables. The maximum correlation coefficient was 0.576, which was less than 0.917 (square root of AVE 0.8408). Therefore, it can be indicated that the discriminant validity of LMWB is satisfactory.

**Table 5:** LMWB’s AVE and CR.

Variable	Factor loading									
LMWB	0.954	0.951	0.948	0.948	0.938	0.937	0.910	0.889	0.871	0.813
CR	0.9814									
AVE	0.8408									

#### 3.3.3 Study 3c: nomological network, predictive validity, and incremental validity

In this study, we examined predictive validity and incremental validity and established the nomological network of LMWB. We investigated trust, performance, work well-being, emotional exhaustion, and turnover intention as theoretically relevant variables for LMWB. Specifically, trust refers to one party’s positive expectations of the other party’s actions and intentions, as well as their willingness to bear harm from the other party. A high degree of trust stems from a positive cooperative relationship between the two parties (Zhao et al., 2024). While LMWB is a typical behavior in which leaders control subordinates’ minds to achieve their goals, leaders’ self-interest reduces employees’ trust in the leader. Performance is used to assess an employee’s current performance and future promotions (Goodman and Svyantek, 1999). LMWB means that leaders suppress their subordinates and control their future development for personal gain, making it impossible for employees to handle their current work better and effectively utilize the information-processing capabilities related to their own development, thereby leading to a decline in work performance. Similarly, job well-being includes job satisfaction and job-related emotions (Zhao, 2014). LMWB obviously brings negative emotions to employees, thereby leading to a decrease in their own job well-being. In addition, the self-interested behavior of leaders also affects the psychological resources of employees, causing them to be emotionally exhausted and subsequently leading to a tendency to resign. Finally, we assessed the predictive and incremental validity of LMWB.

##### 3.3.3.1 Participants

We used data from Sample 3b in Study 3c. Additional variables were collected in this study that were not discussed earlier in Study 3b. Additional variables are detailed below.

##### 3.3.3.2 Measures

In Study 3c, the Likert five-point scoring method was used for the measurement (1 = strongly disagree, 5 = strongly agree). Only work performance was filled out by the leaders, and the rest were filled out by the employees.

###### 3.3.3.2.1 Trust (T2)

The trust measurement used the 10-item scale developed by Li and Yan (2007); an example item is “During contact with the direct leader, I can freely communicate my thoughts and emotions with him.” The Cronbach’s *α* coefficient was 0.98.

###### 3.3.3.2.2 Performance (T2)

The work performance measurement used the 9-item scale developed by Goodman and Svyantek (1999); an example item is “This employee can achieve work goals through planning and organization and can complete the work before the deadline.” The Cronbach’s *α* coefficient was 0.93.

###### 3.3.3.2.3 Work well-being (T2)

The work well-being measurement used the 6-item scale developed by Zheng et al. (2015); an example item is “Overall, I am very satisfied with the work I am doing.” The Cronbach’s *α* coefficient was 0.94.

###### 3.3.3.2.4 Emotional exhaustion (T2)

The emotional exhaustion measurement used the 3-item scale developed by Watkins et al. (2014). An example item is “When thinking of having to face another day of work, I feel exhausted.” The Cronbach’s *α* coefficient was 0.90.

###### 3.3.3.2.5 Turnover intention (T2)

The turnover intention measurement used the 4-item scale developed by Mobley et al. (1978); an example item is “I often feel bored and want to change to a new unit for the current job.” The Cronbach’s α coefficient was 0.73.

##### 3.3.3.3 Results

###### 3.3.3.3.1 Nomological network

The correlation coefficients of these variables are shown in Table 6. Consistent with the predictions in the previous chapters, LMWB was significantly negatively correlated with employee work performance (*r* = −0.176, *p* < 0.01), employee trust (*r* = −0.402, *p* < 0.01), and employee perceived work well-being (*r* = −0.239, *p* < 0.01) and was significantly positively correlated with employee emotional exhaustion (*r* = 0.610, *p* < 0.01) and employee turnover intention (*r* = 0.418, *p* < 0.01).

**Table 6:** Correlation analysis of variables in nomological network.

Variable	M	SD	1	2	3	4	5	6
1. LMWB	4.132	1.667	1					
2. Performance	4.040	0.558	−0.176^**^	1				
3. Trust	3.474	1.037	−0.402^**^	−0.050	1			
4. Work well-being	3.647	0.840	−0.239^**^	0.113	0.222^**^	1		
5. Emotional exhaustion	3.334	1.008	0.610^**^	−0.116	−0.171^**^	−0.090	1	
6. Turnover intention	2.716	0.608	0.418^**^	−0.196^**^	−0.297^**^	−0.242^**^	0.487^**^	1

*N* = 256, ^**^p < 0.01, ^*^p < 0.05, M = Mean, SD = Standard Deviation.

###### 3.3.3.3.2 Predictive validity

First, based on the results of the nomological network in the previous section, it can be seen that LMWB is significantly negatively correlated with employee work performance, employee trust, and employee work well-being and is significantly positively correlated with employee emotional exhaustion and employee turnover intention. Therefore, we conducted a regression analysis of 256 responses. The specific steps are as follows: The first step is to input control variables, including employee gender, age, education level, and working years; the second step is to input LMWB, and the results shown in Table 7 confirm the predictive validity of LMWB.

**Table 7:** LMWB’s predictive validity.

Variable	Performance		Trust		Work well-being		Emotion exhaustion		Turnover intention	
	1	2	1	2	1	2	1	2	1	2
Gender	0.046	0.050	−0.24	−0.016	0.165^**^	0.170^**^	0.054	0.042	0.012	0.004
Age	0.170^*^	0.145	0.081	0.023	0.095	0.061	−0.341^**^	−0.253^**^	−0.261^**^	−0.202^**^
Education	0.064	0.066	−0.137^*^	−0.133	−0.191^**^	−0.189^**^	−0.219^**^	−0.225^**^	−0.078	−0.081
Work year	0.010	0.035	−0.120	−0.062	−0.048	−0.014	0.105	0.017	0.144^*^	0.085
LMWB		−0.170^**^		−0.392^**^		−0.231^**^		0.596^**^		0.400^**^
*R* ^2^	0.033	0.061	0.032	0.182	0.067	0.119	0.102	0.450	0.049	0.205
△*R^2^*	0.033	0.028^**^	0.032	0.150^**^	0.067	0.052^**^	0.102^**^	0.347^**^	0.049^*^	0.156^**^

*N* = 256, ^**^p < 0.01, ^*^p < 0.05.

###### 3.3.3.3.3 Incremental validity

In order to better examine the incremental effect of LMWB, this section adopts the stepwise regression method for analysis. In the first step, control variables, such as gender, age, education level, and working years, were included. In the second step, abusive supervision and exploitative leadership were included. Third, we added LMWB. The purpose of these operations is to examine whether LMWB has an incremental effect on work performance, trust, work well-being, emotional exhaustion, and turnover intention after controlling for demographic variables and other leadership behavior variables. According to Table 8, the results showed that LMWB had an incremental effect among these variables, which proves that it is an important supplement to other types of leadership behavior.

**Table 8:** LMWB’s incremental validity.

Variable	Step	Performance	Trust	Work well-being	Emotion exhaustion	Turnover intention
Abusive supervision	1	0.033	0.032	0.067	0.102	0.049
	2	0.016^*^	0.110^**^	0.002	0.382^**^	0.253^**^
	3	**0.014** ^**+**^	**0.057** ^**^	**0.063** ^**^	**0.081** ^**^	**0.017** ^*^
Exploitative leadership	1	0.033	0.032	0.067	0.102	0.049
	2	0.013	0.136^**^	0.003	0.402^**^	0.243^**^
	3	**0.016** ^*^	**0.046** ^**^	**0.059** ^**^	**0.075** ^**^	**0.018** ^*^

The value of the first step is *R*^2^, and the values of the second and third steps are △*R*^2^; *N* = 256. ^+^*p* < 0.1, *p < 0.05, ^**^p < 0.01. The bolding indicates the correlation and significance between the variables.

## 4 Discussion

The present research introduces the construct of LMWB, develops and validates a scale to measure it, and examines its relationship with other key constructs. LMWB represents a leader’s conscious attempt to alter a subordinate’s perception of reality and self-concept to achieve mental control and fulfill selfish goals. Consistent with this conceptualization, EFA demonstrated that LMWB is a unidimensional construct, and CFA confirmed this. We also demonstrated the incremental predictive validity of LMWB for performance, trust, work well-being, emotional exhaustion, and turnover intention. Collectively, our work reveals the usefulness of LMWB as a predictor of employee attitudes and behaviors and the value of our measure to operationalize it.

### 4.1 Theoretical contributions

This study makes several contributions to leadership literature. First, introducing the LMWB construct extends our theoretical understanding of different leadership behaviors in meaningful ways. Although LMWB is similar to other destructive leadership behaviors in organizations (such as abusive supervision and exploitative leadership), they all have serious negative impacts on the survival and development of the organization; there still exist significant differences. Previous research has mainly focused on overt hostility (such as abusive supervision; Tepper, 2000) or resource exploitation (such as exploitative leadership; Schmid et al., 2019), while ignoring the fact that indirect and covert aggression is also a unique and equally destructive form of interpersonal harm (Björkqvist, 1994). The establishment of the LMWB construct formally introduces these subtle, micro-level interactional dynamics into the organizational leadership literature. This enables an empirical examination of the manipulative experiences reported by 50.8% of employees (QQ News, 2024), which cannot be fully captured by existing theoretical frameworks. Second, drawing from the literature on interpersonal manipulation and the gaslighting effect, we provide a robust theoretical explanation for how LMWB causes harm—namely, by systematically distorting a subordinate’s perception of reality and self-awareness, thereby creating an internal conflict. This theoretical linkage to psychological abuse literature enriches our understanding of the mechanisms underpinning subtle workplace harm. Third, the scale adhered to the most rigorous paradigms and was cross-validated for structure using different samples. We believe that it is necessary to explore LMWB and develop a scale in order to provide tools for further research on its formation and functional mechanisms. The scale developed currently not only expands the application of LMWB but also opens up a path for the quantitative measurement of LMWB, facilitating subsequent research.

### 4.2 Practical contributions

These findings have valuable implications for practitioners. First, we found that LMWB was significantly negatively correlated with employees’ work performance, trust, and work well-being and significantly positively correlated with emotional exhaustion and turnover intention. Therefore, it is necessary to supervise the “manipulative” aspects of the talent management process, avoid the occurrence of LMWB, encourage employees to actively raise questions, and improve their own capabilities to create value for the organization. Second, given the chain effect of destructive leadership behaviors within the organization (Liu et al., 2012), in order to curb the spread of leadership problems at the top level to the middle level, organizations can also train or select their leaders to reduce their manipulations toward employees and listen more to their opinions and ideas. Furthermore, through the definition and scale development in this study, operational tools were provided for enterprises to identify and address LMWB. For example, organizations can incorporate the LMWB scale into leadership development programs and 360-degree feedback systems to help leaders become aware of potentially manipulative behavioral patterns. By identifying such behaviors early, organizations can provide targeted coaching and training to promote healthier leader-subordinate relationships and foster a positive organizational climate.

### 4.3 Limitations and directions for future research

This study has several limitations that should be noted and serve as a starting point for future research. First, LMWB developed in this study has a certain universality in the workplace context, but it distinguishes the subject, that is, the manipulation of leaders toward subordinates. However, the manipulation behavior does not necessarily flow from top to bottom. Future research could extend this line of inquiry in two directions: a horizontal perspective, examining manipulation among peers (employee-to-employee), and an upward perspective, investigating how employees manipulate their leaders. In addition, the scale developed in this study has a strong subjective judgment, and future research can focus on observable specific behaviors for further exploration. Second, the variable data measurement in this study mainly adopts self-report. While self-reporting is appropriate for capturing employees’ subjective perceptions of leader manipulation, it may be susceptible to common methods and attributional biases. Future research could strengthen the validity of these findings by incorporating multi-source designs (e.g., peer or third-party ratings) and behavioral observation methods. Additionally, during the content validation process, item-level classification accuracy was not systematically recorded, and future research could incorporate formal misclassification metrics to further strengthen discriminant validity at the content level. Third, this study focused on workplaces in China. The validation sample consisted mainly of highly educated employees from developed urban areas, which limits the immediate generalizability of the findings to less-educated employees or those in small- and medium-sized cities. Different countries, educational levels, and regional contexts may lead to different perceptions of manipulation, and the current items may not function equivalently across diverse populations. Future research could test measurement invariance across these groups and, where necessary, adapt the scale to ensure its applicability in broader workplace settings.

## 5 Conclusion

In this study, we advance the concept of LMWB and develop and validate a scale to measure it. We examine the nomological network of LMWB and demonstrate its incremental predictive validity for work outcomes. Providing a measurement tool for LMWB will enable an expanded body of research on this unique construct, which stands to enhance our awareness of the manipulation behaviors of leaders toward their subordinates.

## Data Availability

The raw data supporting the conclusions of this article will be made available by the authors, without undue reservation.

## Appendix A

### 19 LMWB items

1. Intentionally changing orders frequently to make you obey him/her rather than improving your work performance.
2. Deliberately denying and suppressing you to make you follow his/her orders.
3. Intentionally belittling you with words to intensify your sense of incompetence and make you obedient.
4. Deliberately praising others to highlight your uselessness and make you obey.
5. Intentionally magnifying your work mistakes to intensify your sense of guilt and make you obedient.
6. Deliberately assigning tasks beyond your ability to make you unable to complete them and thus have to follow him/her.
7. Interacting with you in a way that is alternately cold and warm to create a sense of insecurity and make you obedient.
8. Deliberately emphasizing his/her importance to you to make you follow.
9. Intentionally suppressing your independent thinking to cultivate obedience.
10. Deliberately instilling his/her thoughts into you to unconsciously change your cognition.
11. Intentionally seeking out flaws in your thinking to make you follow him/her.
12. Deliberately blocking effective information to make you dependent on him/her.
13. Making empty promises in your work to make you follow his/her orders.
14. Deliberately sabotaging your relationships with others to isolate you and make you ultimately dependent on him/her.
15. Deliberately showing hypocritical concerns to make you feel grateful and willing to obey him/her.
16. Deliberately emphasizing his/her power and status to make you obey him/her.
17. Threatening and intimidating you to make you have no choice but to obey.
18. Intentionally setting up a persona or label for you to make you act in accordance with it.
19. Deliberately flattering you to make you misjudge yourself and achieve the goal of making you regress or fail.

## Appendix B

### 10 LMWB items

1. Deliberately emphasizing his/her importance to you to make you follow.
2. Deliberately instilling his/her thoughts into you to unconsciously change your cognition.
3. Intentionally seeking out flaws in your thinking to make you follow him/her.
4. Making empty promises in your work to make you follow his/her orders.
5. Deliberately sabotaging your relationships with others to isolate you and make you ultimately dependent on him/her.
6. Deliberately showing hypocritical concerns to make you feel grateful and willing to obey him/her.
7. Deliberately emphasizing his/her power and status to make you obey him/her.
8. Threatening and intimidating you to make you have no choice but to obey.
9. Intentionally setting up a persona or label for you to make you act in accordance with it.
10. Deliberately flattering you to make you misjudge yourself and achieve the goal of making you regress or fail.
